# One-Piece Zirconia Ceramic versus Titanium Implants in the Jaw and Femur of a Sheep Model: A Pilot Study

**DOI:** 10.1155/2016/6792972

**Published:** 2016-12-12

**Authors:** A. Siddiqi, W. J. Duncan, R. K. De Silva, S. Zafar

**Affiliations:** ^1^School of Dentistry and Health Sciences, Charles Sturt University, Orange, NSW 2800, Australia; ^2^Sir John Walsh Research Institute, Faculty of Dentistry, University of Otago, Dunedin, New Zealand; ^3^Discipline of Paediatric Dentistry, School of Oral Health, University of Western Australia, Perth, WA, Australia

## Abstract

Reports have documented titanium (Ti) hypersensitivity after dental implant treatment. Alternative materials have been suggested including zirconia (Zr) ceramics, which have shown predictable osseointegration in animal studies and appear free of immune responses. The aim of the research was to investigate the bone-to-implant contact (BIC) of one-piece Zr, compared with one-piece Ti implants, placed in the jaws and femurs of domestic sheep. Ten New Zealand mixed breed sheep were used. A One-piece prototype Ti (control) and one Zr (test) implant were placed in the mandible, and one of each implant (Ti and Zr) was placed into the femoral epicondyle of each animal. The femur implants were submerged and unloaded; the mandibular implants were placed using a one-stage transgingival protocol and were nonsubmerged. After a healing period of 12 weeks, %BIC was measured. The overall survival rate for mandibular and femur implants combined was 87.5%. %BIC was higher for Zr implants versus Ti implants in the femur (85.5%, versus 78.9%) (*p* = 0.002). Zirconia implants in the mandible showed comparable %BIC to titanium implants (72.2%, versus 60.3%) (*p* = 0.087). High failure rate of both Zr and Ti one-piece implants in the jaw could be attributed to the one-piece design and surface characteristics of the implant that could have influenced osseointegration. Further clinical trials are recommended to evaluate the performance of zirconia implants under loading conditions.

## 1. Introduction

Implant dentistry using titanium dental implants has revolutionized the treatment of partially and fully edentulous patients [1]. However, there is also increasing awareness that all dental biomaterials release substances and can affect the oral environment to varying degrees and may also contribute to local allergic reactions [2, 3]. Commercially pure titanium has been commonly employed for the manufacture of implants and implant-abutments due to its biocompatibility, high corrosion resistance, and good mechanical properties [4]. However hypersensitivity to titanium may be more frequent than previously thought [5, 6]. Reports have documented titanium hypersensitivity due to dental implants and some reports have implicated titanium hypersensitivity as a potential factor in dental implant failure [7–9]. Frisken and colleagues [10], in a sheep model, observed elevated titanium levels in lymph nodes following aseptic loosening of oral implants and concluded that an elevated concentration of metal ions might act as a local immunosuppressant, analogous to reports for aseptic loosening of orthopaedic implants. This is supported by case reports of titanium particles in the peri-implant soft tissues of human patients [11].

Even though titanium has been used as a biomaterial for more than 50 years, several reports have identified its potential toxicity [12]. Recently, Sakellariou and colleagues [13] reported postoperative spinal infection due to titanium spinal implants. Similarly, Hettige and Norris [14] documented a case of mortality after a suspected fatal local allergic response of the brain to a titanium cranioplasty. Patients sensitive to metals such as nickel, aluminium, or cobalt appear to be more susceptible to titanium hypersensitivity reactions, and a more vigilant approach should be adopted and special care should be taken in the selection of the implant biomaterial for these patients [15].

One of the zirconia compounds is Yttria-stabilized tetragonal Zirconia-polycrystal (Y-TZP), which has been suggested as an alternative to titanium as it has similar biocompatibility and mechanical properties and has a more aesthetically acceptable colour than the metallic grey of titanium [16, 17]. Zirconia has been reported to be highly biocompatible with no local or systemic toxic effects after implantation [18, 19]. Finite Element Analysis (FEA) modelling suggests that Y-TZP implants are able to sustain chewing stresses and have a similar stress distribution to commercially pure titanium [20, 21]. Zirconia is a relatively new implant material compared with titanium [6, 17].

The use of animal models is an essential step in the testing of new biomaterials prior to use in humans. There are many animal models for dental implant research, each having differences in bone remodelling and bony architecture, with potential advantages and disadvantages [22]. Sheep have been used as an animal model for various fields of biomedical research [23–26]. A mandibular oral implant model using domestic sheep has been investigated [24]. Other researchers have established extraoral models for implant research in sheep, including tibia, lower femur, and maxillary sinus [25, 26]. Simultaneous implant placement into multiple sites in sheep allows us to compare osseous healing in different bone types (trabecular cancellous bone or dense cortical bone) modelling different sites and bone quality in human patients [27].

The majority of researchers have used rabbits and minipigs to evaluate zirconia implants [19, 22]. Zirconia showed similar % BIC to that of Ti implants in most of the studies [28–30]. Studies using preclinical experimental models have conducted histology at different time points to provide a sequential picture in the healing process that leads to a stable interface between soft and hard tissue and implant biomaterial. The aim of the current research was to assess the osseointegration of one-piece zirconia implants, compared with one-piece Ti implants, placed simultaneously in the jaws and femurs of sheep model at a predetermined time point (12 weeks of healing). This is supported by the fact that a bony defect in sheep will completely heal in 12 weeks [31] and this corresponds to 16 weeks of healing in humans. Four months is a commonly used healing period in humans for implants, which is why we used the equivalent period in our sheep study. Furthermore, the trabecular bone density in sheep is reported 1.5–2 times greater than that of humans [32, 33].

## 2. Methodology

**Figure 1:**
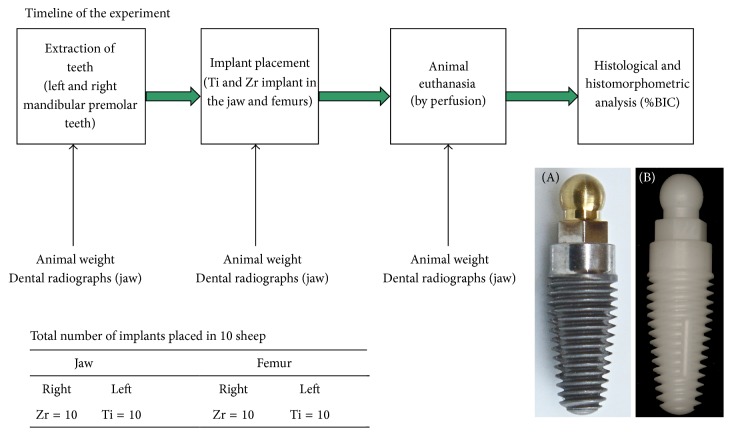
Prototype one-piece implants. (A) One-piece Ti implant. (B) One-piece Zr implant.

Ten adult New Zealand mixed breed female sheep aged 4-5 years and with average weight of 65 kg were used in this trial. The study was approved by the Animal Ethic Committee, University of Otago, Dunedin, New Zealand. The ARRIVE guidelines for preclinical* in vivo* studies were strictly followed throughout the study. Each sheep received one Ti (control) and one Zr (test) implant in the mandible (right and left) and in the femoral epicondyle (right and left) (Figure 1). One-piece prototype zirconia implants (commercially not available) of matching design were manufactured by Southern Implants® (Irene, South Africa) for the research. These implants were tapered in profile with a threaded implant body, a transmucosal cylindrical collar, and a ball-abutment (Figure 1). The implants were roughened using an acid-etching technique (*R*_*a*_ values between 0.5 and 0.8 *μ*m). The implants were Ø3.8 mm in diameter and 10 mm in length for the mandible and Ø5 mm in diameter and 10 mm in length for the femur. Ball abutments were 3.1 mm in diameter for wider implants (5 mm) and 2.25 mm for the standard diameter implants (3.8 mm). Implants were placed in the mandibular premolar sites and in the femurs of each sheep.

## 3. Anaesthesia Technique for All Surgical Procedures

The animal surgeries were carried out at the Department of Laboratory Animal Services at the University of Otago, New Zealand. The sheep were weighed before the commencement of the anaesthesia. Sheep were fasted prior to surgery and preoperative antibiotics were administered (penicillin/streptomycin 3 mL/kg IM). Anaesthesia was induced with thiopentone 20 mg/kg i.v., and an endotracheal tube was passed orally. General anaesthesia was maintained with halothane (1-2% to effect) and nitrous oxide/oxygen in a ratio of 2 : 1.

### 3.1. Stage I (First Surgery): Dental Extractions

**Figure 2:**
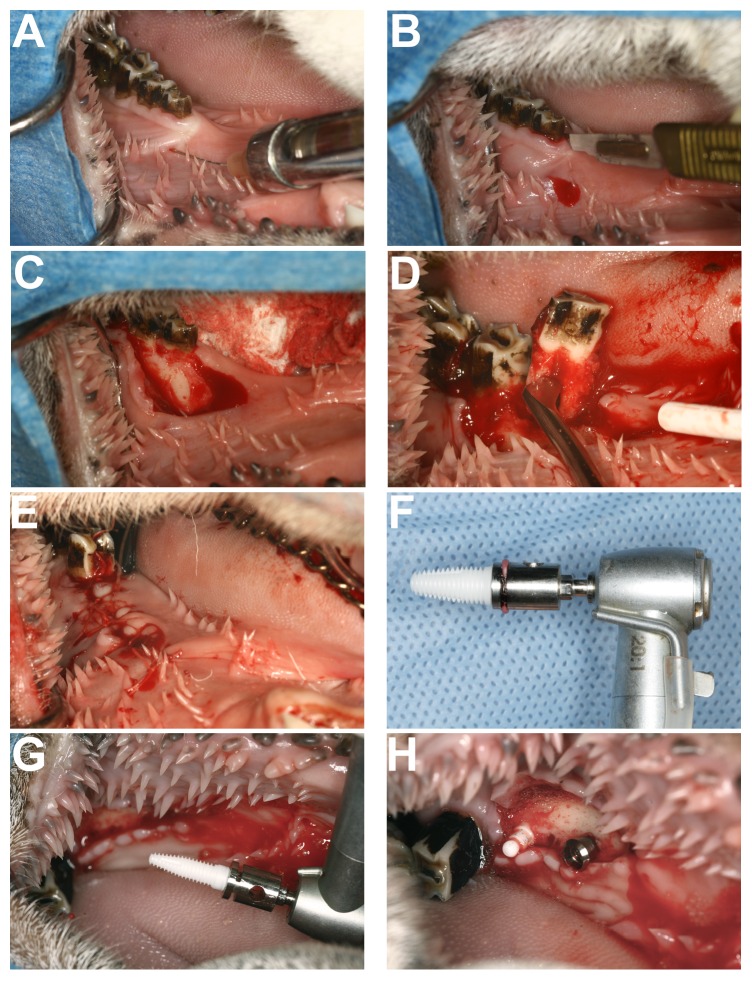
Sheep jaw surgery. (A) Infiltration of the local anaesthetic solution. (B) Relieving incision on the alveolar crest for better access during extraction. (C) Full thickness-periosteal flap reflected for extraction. (D) Tooth extraction using elevator. (E) Closure of the wound with 3/0 vicryl. (F and G) One-piece Zr in the implant drill. (H) One-piece Ti and Zr implants in the jaw.

An atraumatic approach for the extraction of the teeth was adopted. Under general and local anaesthesia, a mucoperiosteal flap was raised around the three mandibular premolars on each side of the lower jaw and the teeth were loosened with periotomes and elevators and then sectioned with a tungsten carbide tapered fissure bur and removed in pieces. The oral wounds were closed with resorbable Dexon™ 3/0 sutures (Ethicon, Inc. Somerville, New Jersey) (Figures 2(A)–2(E)). Dental radiographs of both sides of lower premolar area were taken in order to confirm any root remnants. Postoperatively, Savacol® (chlorhexidine, 10 cc 0.2% aq) solution was applied daily to the surgical sites, starting following the day of surgery, for three days, and the sheep were returned to the farm.

### 3.2. Stage II (Second Surgery): Implant Placement in the Jaw and Femur

After 12 weeks of healing following dental extractions, the sheep were returned to the Department of Laboratory Animal Services (HercusTaieri Resource Unit – HTRU, University of Otago, New Zealand) and starved, anaesthetized and prepared as before.

*Jaw Surgery.* A standard surgical procedure for implant placement was used under sterile conditions at all times. A flap was raised in the healed edentulous ridge from which the mandibular premolar teeth were removed, and one implant on each side of the jaw per sheep was placed accompanied by irrigation with copious chilled saline to prevent overheating (Figures 2(F)–2(H)). The mandibular implants were 3.8 mm diameter and 10 mm long and were either one-piece Ti or one-piece Zr. Dental radiographs were taken to check implant position and peri-implant bone.

**Figure 3:**
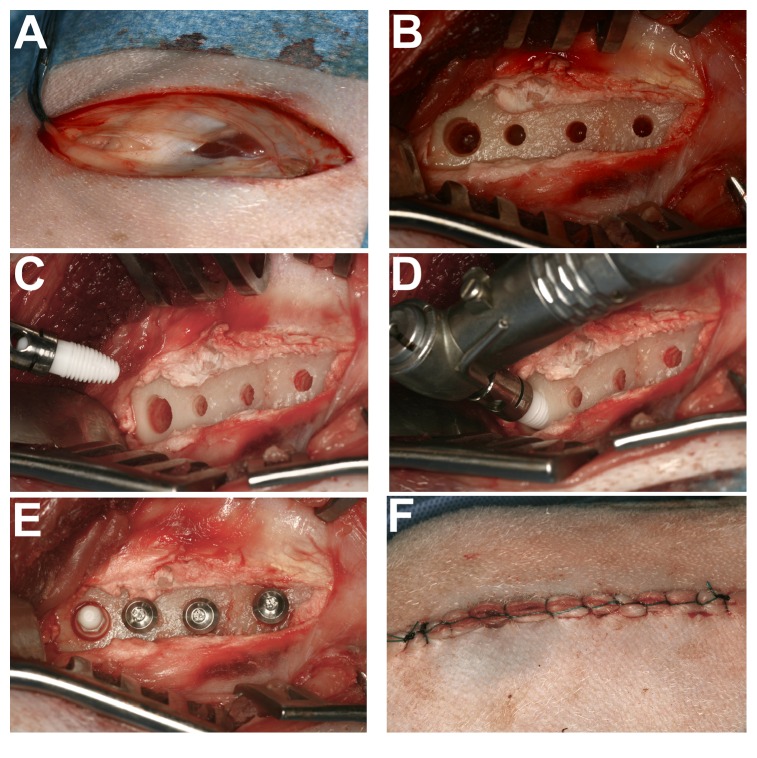
Sheep femur surgery. (A) Incision and reflection of the overlying skin and subcutaneous tissues at the femur implant site. (B) Preparation of the implant site. (C–E) Surgical placement of a wide diameter zirconia and titanium implant. (F) Skin closure in layers after implant placement. The other three implants will be reported elsewhere.

*Femur Surgery.* The surgical approach used the femoral dental implant model discussed by Chappard and colleagues [25] as modified by Duncan, 2005 [24]. The hind legs were shaved and the skin was disinfected with iodine and alcohol. Each femur was exposed by a classic medial approach from great trochanter to distal epiphysis* via* a skin incision of 6–8 cm in length. The periosteum was incised and raised as a two-sided flap. An osteotomy was prepared and implant surgery drill sequence was followed according to the manufacturers' instructions. Pretapping of the implant socket was not performed. One implant was placed in each of the femoral distal metaphyses at low speed, with copious chilled saline irrigation (Figure 3). Thus each animal received two of the one-piece implants, one into each femur. Additional implants were also placed during this surgery into the femoral sites but will not be reported further here.

#### 3.2.1. Animal Euthanasia for Implants Retrieval

After a healing period of three months, the animals were euthanized under general anaesthesia with an overdose of thiopentone. The animals were then perfused via the carotid arteries with heparinized saline followed by formalin fixative (1L chilled fresh 10% paraformaldehyde per side). Mandibular and femoral* en bloc* resections were retrieved and further fixed in formaldehyde. A block of bone containing each implant was then dissected out with fine handsaws. Formalin-fixed specimens from mandibular and femoral sites were reduced to approximately 1 cm × 1 cm × 2 cm in height and rinsed in water.

All specimens were dehydrated and embedded in methyl-methacrylate at approximately 10°C. Specimens were then sectioned longitudinally using an R330 diamond wheel on a Struers Accutom-50® precision cut-off saw (Intellection Pty Ltd, Australia). Sections were then prepared to a final grit size of 4000 and final section thickness of 80–100 *μ*m was prepared. The slides were stained with a solution containing one part MacNeal's tetrachrome (methylene blue, azur II, and methyl violet) and two parts toluidine blue. Sections were viewed using an Olympus Vanox-T microscope at 2x magnification (Olympus Australia Pty Ltd, Australia) and digital images were captured using a Diagnostic Instruments SPOT RT Colour camera (SciTech Pty Ltd, Australia).

**Figure 4:**
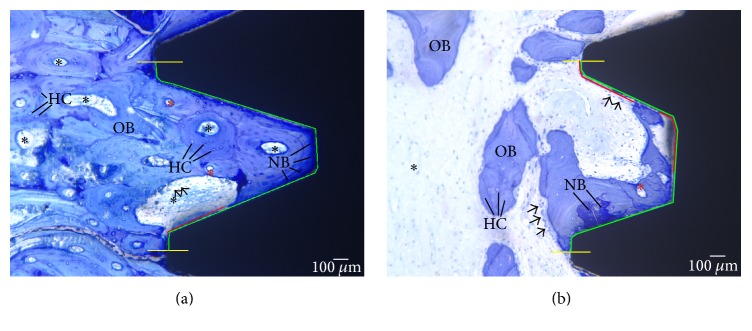
Measurement of bone-to-implant contact using NIH image analysis software, ImageJ®. Histological image of titanium implant in sheep femur (4x). Green line represents the total area of the implant. Red line represents the implant thread area not immediately in contact with the bone. So % BIC: green-red.

Histomorphometric analysis of bone-to-implant interface can be measured in various ways including thread volume fill (volume of bone found within an implant thread), % of bone-to- implant contact (% BIC) on the implant surface of the entire implant, or the % BIC of the “three consecutive best threads.” Histomorphometric analysis of the three best threads has been a well-documented method of measuring osseointegration [25]. The study analysed % BIC using a “best-three consecutive threads” technique and compared zirconia with titanium implants placed into the jaw and femurs of sheep. Two sections per implant were analysed. Images of the histological sections were digitized at 2x and 4x magnification and the % BIC contact was quantified histomorphometrically using NIH Image analysis software, ImageJ (ImageJ - Research Services Branch, NIH, Bethesda, MD, USA and Rasb and & ImageJ, 1997–2012) (Figure 4).

### 3.3. Statistical Analysis

The data were analysed using GraphPad PRISM® software (version 5.04, La Jolla, CA, USA). A pair-wise comparison between zirconia (test group) and titanium (control group) was performed. Wilcoxon signed rank was used for nonparametric comparison of nominal, nonnormal paired data.

## 4. Results

### 4.1. Stage I (First Surgery): Dental Extractions

The anaesthesia and the extraction of the mandibular premolars were uneventful, and all sheep recovered well. No postoperative complications were observed during the first week of healing and the animals were returned to the farm until the second stage of surgery (implant placement) and no significant changes in the weight were noted.

### 4.2. Stage II (Second Surgery): Implant Placement in the Jaw and Femur

Animals were weighed before anaesthesia to note any significant changes in the eating patterns after the removal of the mandibular premolars. No significant changes in the weight were observed. Four extraction sockets remained unhealed and/or infected (4/20), so implants were placed distal to these sites to prevent infection at the implant site.

## 5. Euthanasia and Postmortem

All animals survived jaw and femur (implant) surgery and were available for evaluation. Clinical evaluation of the mandibular implants revealed that three implants were lost, while two implants were loose (Table 1). The overall mandibular implant survival rate was 75%. No local infection or pathology was noted at the femur implant sites. All femur implants were osseointegrated and were clinically stable with a 100% survival rate. An overall survival rate (combining mandibular and femur implants) of 87.5% was observed in this trial, with 100% for all titanium implants and 83.3% for all zirconia implants.

## 6. Histological Description of Integration

The histological images were classed into four categories: “integrated” (the first bone-implant contact was at the first thread and the implant was well integrated for the entire length), “failing” (the first bone-implant contact did not occur at the first thread although more apical parts were integrated), “not integrated” (the implant is surrounded with fibrous connective tissue), and “lost” (the implant had been lost at postmortem).

## 7. Integration of Mandibular Implants

Gross histological evaluation of the Ti mandibular implants showed 7/10 implants were integrated, while the other three were failing. On the other hand, 3/10 Zr implants had failed and been lost, 2/10 were not integrated, one was failing, and only 4/10 could be considered successfully integrated. No statistical significant difference was found (*p* = 0.18). Overall bone-implant integration was 11/20 = 55%.

## 8. Integration of Femur Implants

All implants appeared integrated in the femur. Condensation of bone was observed around the unloaded femoral implants, which has been described previously in trabecular bone [34] and which we have previously observed in implants placed into the femur of sheep [35]. The condensation was categorized as “minimal,” “some” (extending around part of the periphery of the implant), or “considerable” (a dense layer of bone had formed around the entire periphery of the implant). Twenty percent of the Ti implants had minimal bone condensation, 3/10 had some condensation, and 5/10 had considerable bone condensation and increased density around the entire periphery. On the other hand, only one Zr implant had some condensation, whereas 90% had considerable bone condensation and increased density around the entire implant periphery. Some variability in the size of marrow spaces and number of trabeculae was noted, with 4/10 Ti showing smaller marrow spaces and all Zr being surrounded by larger spaces. There were no statistically significant differences when the categories for condensation were codified and compared (*p* = 0.15).

## 9. Histomorphometric Analysis

**Figure 5:**
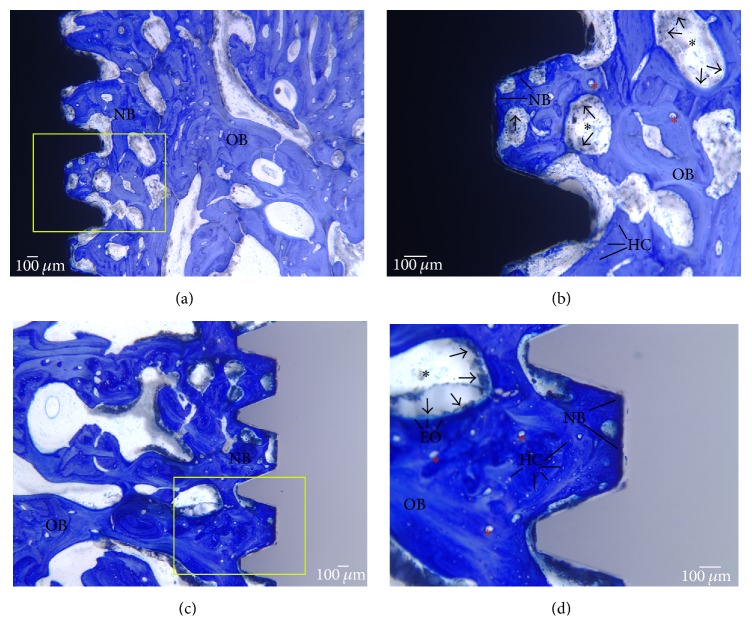
Histology images of sheep jaw stained with MacNeal's tetrachrome at 12 weeks of healing. (a) Titanium implant at 2x. (b) Titanium implant at 4x. (c) Zirconia implant at 2x. (d) Zirconia implant at 4x. Old mineralized bone (OB). New bone (NB), unmineralized bone. Arrows represent the lining of osteoblast cells with osteogenic potential. Immediately following the deposition of osteoid, multinucleated osteoblasts commence the remodelling process and the formation of a Haversian system. Osteoid undergoes maturational changes that prepare them for the initial deposition of calcium phosphate crystals called mineralization front. Osteons (red asterisks) represent the structural end result of a focus of bone remodelling. Black asterisks represent hematopoietic and fatty marrow. Haversian canals (HC) enclose vascular structures, nerves, and lining cells. The development of primary and secondary osteons representing active bone formation and high BIC is visible in the figure. Scale bar: 100 *μ*m.

**Figure 6:**
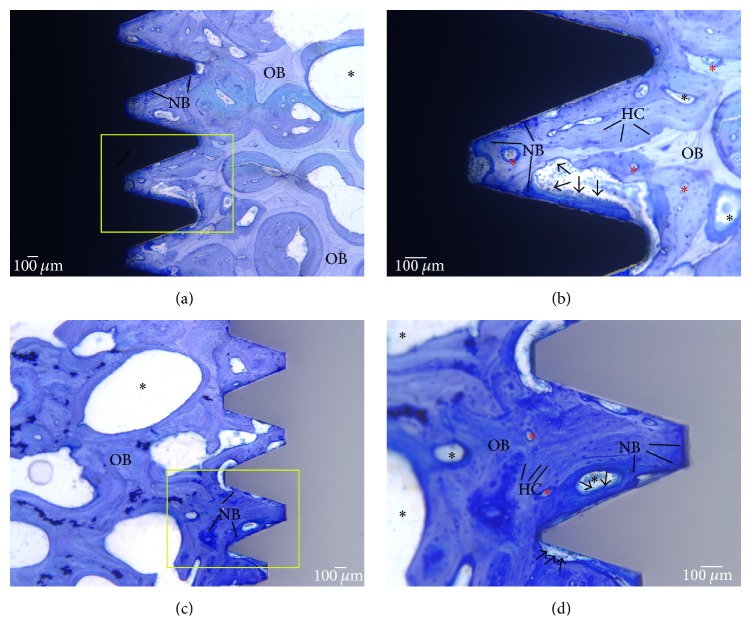
Histology images of sheep femur under light microscope after 12 weeks of healing. (a) Titanium implant at 2x. (b) Titanium implant at 4x. (c) Zirconia implant at 2x. (d) Zirconia implant at 4x. Old bone (OB), mineralized. New bone (NB), unmineralized bone. Arrows represent the lining of osteoblast cells with osteogenic potential. Immediately following the deposition of osteoid, multinucleated osteoblasts commence the remodelling process and the formation of Haversian system. Osteons (red asterisks) represent the structural end result of a focus of bone remodelling. Black asterisks represent hematopoietic and fatty marrow. Haversian canals (HC) enclosing vascular structures, nerves, and lining cells. Dense bone with high BIC can be seen in (d). Scale bar: 100 *μ*m.

For implants placed into the femur, statistically significant differences were observed between the % BIC of Zr and Ti implants (Table 2). The survived Zr implants showed greater % BIC (0.002). With respect to the implants placed into the mandible, five of ten Zr mandibular implants failed to osseointegrate and one was failing. After excluding the failed zirconia mandibular implants from the data, the remaining zirconia implants showed comparable % BIC to the titanium implants (*p* = 0.087). When the results from the two different surgical sites are considered, bone-implant contact for zirconia implants was 85.5% (SD 14.1) in the femur and 72.2% (SD 23.7) in the mandible. For titanium implants, % BIC was 60.3% (SD 22.4) for the mandible (Figure 5) and 78.9% (SD 18.5) for the femur (Figure 6).

## 10. Discussion

The present study showed successful osseointegration of zirconia and titanium implants in the experimental animals. Zirconia implants showed greater values for % BIC after 12 weeks of healing in the sheep mandible and femur. All submerged implants in the femur showed excellent osseointegration and zirconia implants showed evidence of an influence on the surrounding bone bed. A statistically significant difference was noted in the % BIC of zirconia implants when compared to titanium implants (*p* = 0.002). Similarly in the jaw, zirconia implants also showed greater % BIC compared with titanium implants; however, the difference was not statistically significant, and there was a much higher incidence of negative outcomes, particularly for the one-piece zirconia implants.

In the journey to achieve more predictable osseointegration especially in difficult clinical sites, researchers have attempted to alloy zirconium to titanium [36]. Comparable results have been reported with this new titanium-zirconium alloy compared with traditional commercially pure titanium (cpTi) implants [36]. Recombinant human bone morphogenic proteins-2 (rhBMP-2) gel has also been applied to the zirconia implant surface to enhance local bone formation and the speedy recovery of the prepared implant site for early osseointegration [30]. The researchers found similar % BIC to the zirconia implants with rhBMP-2 gel; however improved healing of the implant site was observed [30].

The % BIC measured in the present research showed slightly higher values of zirconia implants compared with other reported animal studies [28, 29]. We used the sheep femoral site in our study to model maxillary bone in human patients, which is trabecular and cancellous in nature. We chose not to use the sheep tibial site, as this is denser compact bone [36]. Although a number of studies have suggested a tibial model to investigate bone healing around the implanted biomaterials, some studies have also recommended the proximal and distal humerus and proximal and distal femur in sheep model [37, 38]. This could be due to the fact that cancellous bone is biologically more active compared with compact bone and hence is considered as an excellent material to assess bone replacement and induction. To the knowledge of the authors, no research has been conducted using sheep (jaw and femur) as experimental animals to investigate % BIC of one-piece zirconia implants and compared them with one-piece titanium.

The difference in the % BIC reported in multiple studies could be ascribed to the dissimilarities in the biology and structural morphology of the host and ultimately their response to biomaterials.

A number of studies have presented low survival rates of one-piece zirconia implants ranging from 78% to 98% with an observation period of 1–5 years [39, 40]. Most of these studies have shown a trend towards early failure rather than late failure of these one-piece implants. A recent study compared immediately loaded one-piece implants with delayed loaded one-piece and two-piece implants and reported higher bone loss with one-piece implants compared with two-piece implants [41]. However, factors like experimental design of the implant (micro- and macrodesign), surface characteristics and chemistry, surgical protocols, and prosthetic superstructures should also be considered when examining the success or survival of these one-piece implants [42, 43]. Due to the single unit design of one-piece implants and exposure of the supra-mucosal part of the implant head into the oral cavity, a load-free healing period is challenging because of the masticatory and/or tongue movements during function. In the current sheep study the initial loading forces exerted during grazing and tongue movements would have played a role in failure of one-piece Ti and Zr implants in the mandible compared to femur, where such circumstances are not encountered during healing period.

The differences in % BIC could be easily highlighted when comparing femur implants (submerged) with jaw implants (nonsubmerged). The literature has ascribed implant failure to a number of factors including initial stability, poor bone quality (types II and IV), implants placed in heavy smokers, nonsubmerged implants, immediately placed implants, small and/or short implants, inexperienced surgeon, and implants in fresh extraction sockets [44]. Studies have also implicated surface characteristics as an important factor in the successful osseointegration of dental implants and researchers have attempted to enhance this biological process by introducing innovative approaches towards preparation of implant surfaces [45, 46]. In the current study Zr implants were surfaced etched, while Ti implants were sandblasted (not large grit) and acid conditioned. The difference between the surface treatments of Zr and Ti implants in the current study could also have played a role in osseointegration. However, recent literature suggests that moderately rough surfaces can provide optimal clinical outcomes when compared with rougher plasma-sprayed surfaces [46, 47].

The outcomes of this research supported the results of other studies that have demonstrated higher failure rates of one-piece zirconia implants in clinical conditions [48, 49]. One of the limitations of the study was that it observed BIC at only one time point, that is, at 12 weeks. It is acknowledged that the observation of BIC at multiple time points would have provided a complete picture of bone healing in this model with Zr and Ti implants.

## 11. Conclusion

One-piece zirconia implants showed good osseointegration in the sheep femur. However, the high failure rate of both zirconia and titanium one-piece implants when placed into an intraoral site in the lower jaw reflects earlier studies showing poorer results with one-piece implant systems placed using a single-stage protocol. Further clinical trials are recommended to evaluate the performance of zirconia implants under loading conditions.

## Figures and Tables

**Table 1:** Distribution of implants in the jaw and their outcomes.

Mandibular implants					
Implant type	Placed	Integrated	Failing	Not integrated	Lost
Zirconia	10	4	1	2	3
Titanium	10	7	3	0	0

Wilcoxon signed ranks nonparametric comparison of nominal, nonnormal paired data. No stat sig difference, *p* = 0.18. Overall integration was 11/20 = 55%.

**Table 2:** Percentage of bone-to-implant contact around titanium and zirconia implants.

% Bone implant contact	Femur		Mandible	
	Zirconia	Titanium	Zirconia	Titanium
Minimum % BIC	31.4	18.6	12.2	10.6
Maximum % BIC	100	100	100	100
Mean (±SD)	85.5 (±14.1)	79.0 (±18.5)	72.2 (±23.6)	60.2 (±22.4)
